# Editorial: Biomarkers in the era of cancer immunotherapy: zooming in from the periphery to the tumor microenvironment

**DOI:** 10.3389/fimmu.2023.1283186

**Published:** 2023-09-18

**Authors:** Jehad Charo, Bruno Gomes, Kristian Pietras, Arne Östman

**Affiliations:** ^1^ Pharmaceutical Research and Early Development Oncology, Roche Innovation Center Zurich, F. Hoffmann-La Roche AG, Zurich, Switzerland; ^2^ Pharmaceutical Research and Early Development Oncology, Roche Innovation Center Basel, F. Hoffmann-La Roche AG, Basel, Switzerland; ^3^ Department of Laboratory Medicine, Division of Translational Cancer Research, Lund University Cancer Centre, Lund University, Lund, Sweden; ^4^ Department of Oncology-Pathology, Karolinska Institutet, Stockholm, Sweden

**Keywords:** biomarkers, cancer, immunotherapy, imaging, fibroblasts, tumor microenvironment, immune oncology, checkpoint blockade

Cancer immunotherapy (CIT) has become a standard of care in multiple indications. Apart from radically increasing response rate and prolonging survival, CIT offers the ultimate benefit of any therapy, which is a cure (1).

Since the early days of CIT, it was recognized that the benefit is driven by complicated biological mechanisms, which involves a cross-talk between the tumor, its microenvironment, and the overall immune system of the patient (2, 3). Studies aiming to understand and explore CIT mechanisms of action and pharmacodynamic and response prediction biomarkers have initially evaluated each component independently, but ultimately need to look at these questions in concert. Technological advancements in the field combined with the availability of better translational models and ever-expanding clinical datasets enabled basic, translational, and clinical researchers to have a deeper understanding of this complex interaction (4–8).

This combined Research Topic of Frontiers in Immunology and Frontiers in Oncology provides further insight into how multiomics biomarkers from genetic alterations to cellular niche organization can contribute to the outcome of CIT.

Bladder cancer is one of the earliest indications that were shown to be sensitive to CIT. Analyzing genetic and genomic data combined with clinical information from multiple data sets, Xu et al. stratify bladder cancer patients into low- and high-risk groups using a fibroblast growth factor receptor 3 (FGFR3)-related gene signature score. The signature includes components of metabolism and immune signaling genes. Consequently, the low-risk patients with the lower score present with an abundance of immune cell enrichment and are more responsive to CIT, but are less sensitive to certain chemotherapy agents.

The immune landscape, and its prognostic significance, in head and neck squamous cell carcinoma (HNSCC) remains to be better characterized. Baysal et al. present a study combining CIBERSORT-based analyses of prognostic significance of immune cell types in a TCGA cohort, with IHC analyses of an additional cohort. Together, the study identifies novel prognostic associations of CIBER-sort-defined cell types, as well as distinct prognostic IHC-determined immune-regulatory proteins with targeting potential.

Lung cancer is recognized among the most sensitive indications to CIT. Zhao et al. identify a gene expression signature composed of lactic acid metabolism-related genes, which can differentiate lung cancer cell lines from normal bronchial epithelium cells. This signature shows an inverse correlation to survival from three studies, including in response to CIT, correlating positively with tumor mutation burden and mutation rate, but identifies patients with low signature scores and higher TMB as best responders. These data add to the rationale of evaluating lactic acid metabolism targeted CIT (9). Ji et al. explore the association of genomic alterations of development signaling pathway-related genes and response to CIT and identify SMO mutation as correlating with a response but also immune signatures not only in NSCLC cohorts but also in other cancer types.

PD-L1 expression continues to be analyzed regarding its usefulness as a prognostic and/or response-predictive biomarker for CIT. Khan et al. here contribute to this research area with a meta-analysis of publications on metastatic triple-negative breast cancer. The study identifies significant associations between PD-L1 expression and better objective response rate as well as overall survival. The findings emphasize the importance of PD-L1 as a response biomarker and suggest further analyses by including larger randomized studies with non-CIT-treated patients towards better discrimination between the prognostic and predictive nature of PD-L1.

In parallel to continued efforts to use “single/few-marker” approaches for biomarker discovery, there is an emerging field of multi-omics studies. These have the possibility to capture more complex, but also more informative, tumor biology. This approach is represented in the current volume by the study of Wong et al., in multiple myeloma in which patient samples from a phase 1 atezolizumab trial are analyzed. Although preliminary, this study illustrates some of the potential of this type of approach but also emphasizes the need for larger cohorts to obtain more conclusive results.

Combining data from peripheral blood immune cells and patient-matched tumor genetic markers, Dutta et al. explore the potential of predicting response to CIT in non-small cell lung cancer patients. The team identifies baseline memory CD4+ and CD8+ T-cell subsets in the peripheral blood and pathogenic or likely pathogenic mutation in the tumor to be differentially prevalent between responders and non-responders. If validated in a larger dataset, this will be a very interesting approach to identify patients who are more likely to benefit from CIT.

The abundance, maturity, and spatial distribution of tertiary lymphoid structures (TLS) from hematoxylin and eosin (H&E) staining and gene expression data are evaluated by Liang et al. as a prognostic biomarker for CIT response in laryngeal squamous cell carcinoma. The team finds that early non-follicular and follicle-like TLS are associated with immunosuppression and increased immune infiltration, respectively. The maturation of the TLS is associated with higher expression of XCL2, suggesting a cross-talk between immune cell activation and TLS maturation in laryngeal squamous cell carcinoma.

These eight articles touch on a few of the many exciting developments in the field. As in every successful scientific exploration, we have learned more but also identified further questions to be investigated. Extrapolating from that and the fast progress observed in the field, we look forward to exciting developments in the field toward personalizing CIT and maximizing its benefit for the patients.

## Author contributions

JC: Conceptualization, Writing – original draft, Writing – review & editing. BG: Conceptualization, Writing – original draft, Writing – review & editing. KP: Conceptualization, Writing – review & editing. AÖ: Conceptualization, Writing – original draft, Writing – review & editing.
